# Composite Polycaprolactone/Gelatin Nanofiber Membrane Scaffolds for Mesothelial Cell Culture and Delivery in Mesothelium Repair

**DOI:** 10.3390/ijms25189803

**Published:** 2024-09-11

**Authors:** Darshan Tagadur Govindaraju, Hao-Hsi Kao, Yen-Miao Chien, Jyh-Ping Chen

**Affiliations:** 1 Department of Chemical and Materials Engineering, Chang Gung University, Kwei-San, Taoyuan 33302, Taiwan; darshu.tg@gmail.com (D.T.G.);; 2 Division of Nephrology, Chang Gung Memorial Hospital at Keelung, Keelung 20401, Taiwan; kao95812@yahoo.com.tw; 3 School of Medicine, College of Medicine, Chang Gung University, Kwei-San, Taoyuan 33302, Taiwan; 4 Department of Neurosurgery, Chang Gung Memorial Hospital at Linkou, Kwei-San, Taoyuan 33305, Taiwan; 5 Research Center for Food and Cosmetic Safety, College of Human Ecology, Chang Gung University of Science and Technology, Taoyuan 33305, Taiwan; 6 Department of Materials Engineering, Ming Chi University of Technology, Tai-Shan, New Taipei City 24301, Taiwan

**Keywords:** gelatin, scaffold, nanofibers, mesothelial cells, membrane, polycaprolactone

## Abstract

To repair damaged mesothelium tissue, which lines internal organs and cavities, a tissue engineering approach with mesothelial cells seeded to a functional nanostructured scaffold is a promising approach. Therefore, this study explored the uses of electrospun nanofiber membrane scaffolds (NMSs) as scaffolds for mesothelial cell culture and transplantation. We fabricated a composite NMS through electrospinning by blending polycaprolactone (PCL) with gelatin. The addition of gelatin enhanced the membrane’s hydrophilicity while maintaining its mechanical strength and promoted cell attachment. The in vitro study demonstrated enhanced adhesion of mesothelial cells to the scaffold with improved morphology and increased phenotypic expression of key marker proteins calretinin and E-cadherin in PCL/gelatin compared to pure PCL NMSs. In vivo studies in rats revealed that only cell-seeded PCL/gelatin NMS constructs fostered mesothelial healing. Implantation of these constructs leads to the regeneration of new mesothelium tissue. The neo-mesothelium is similar to native mesothelium from hematoxylin and eosin (H&E) and immunohistochemical staining. Taken together, the PCL/gelatin NMSs can be a promising scaffold for mesothelial cell attachment, proliferation, and differentiation, and the cell/scaffold construct can be used in therapeutic applications to reconstruct a mesothelium layer.

## 1. Introduction

The mesothelium was first described in 1827 by Bichat using histological methods, who observed that the serous cavities were bordered by a single layer of flattened cells comparable to those of the lymphatics [1]. This phenomenon has garnered a significant amount of interest in recent years because these cells can regenerate and have a variety of uses in tissue engineering [2]. The mesothelial cells play an important role in mesothelium, as they provide a smooth layer over internal organs, in addition to their many biological and physiological activities. Mesothelial cells, despite their origin from the mesoderm, exhibit characteristics that include both epithelial and mesenchymal cells. The mesothelium layer is generally recognized as a dynamic cellular membrane associated with many important physiological roles, such as fluid and solute transport regulation and immune surveillance [3]. They contribute to several physiological processes, such as secreting growth factors and cytokines, functioning as extracellular matrix (ECM) constituents and promoting tissue repair and regeneration. In addition, they possess anti-inflammatory and immunomodulatory attributes [4]. Peritoneal dialysis may cause structural and functional damage to the peritoneum when exposed to dialysis solutions that have a high glucose content, high molarity, and low pH values. Prolonged contact with these liquids can lead to atrophy of mesothelial cells and sclerosis of the peritoneum [5]. Mesothelial regeneration may occur when cells migrate from the wound’s edge or when mesothelial cells free-floating in the serosal fluid adhere and integrate into the wound surface. However, the establishment of serosal adhesion occurs, which is mostly caused by inadequate healing and cellular transformation. Therefore, the possible use of mesothelial cell transplantation may address the need for patients with chronic renal failure, who suffer from impaired peritoneal dialysis efficacy [6].

In tissue engineering, mesothelial cells offer unique advantages as seeding cells for scaffold-based approaches. Their ability to adhere to various surfaces and secrete bioactive molecules makes them attractive candidates for promoting tissue integration and vascularization within engineered constructs [7]. Moreover, their immunomodulatory properties can mitigate host immune responses, thereby enhancing the biocompatibility and long-term viability of tissue-engineered implants. Recent advancements in biomaterials and scaffold fabrication techniques have facilitated the development of mesothelium-inspired scaffolds capable of mimicking the native tissue microenvironment [8]. These biomimetic constructs leverage the regenerative potential of mesothelial cells to create functional tissues with tailored properties, ranging from simple membranes for wound healing to complex organoids for transplantation. Despite the progress towards harnessing mesothelial cells for tissue engineering applications, several challenges remain, including optimizing cell sourcing, maintaining phenotypic stability in vitro, and promoting controlled differentiation towards desired cell lineages. Addressing these hurdles requires interdisciplinary efforts integrating cell biology, biomaterials science, and tissue engineering principles.

As one of three key components in the tissue engineering triad, the scaffold can interact with seeded cells, which under the induction of growth factors can create a specific environment for cells/tissues to grow systematically [9]. The scaffold can be used to construct an artificial ECM milieu composed of complex fibrous structures such as collagen, glycosaminoglycan, elastin, etc. [10,11]. With its unique structure, the scaffold may provide biochemical or mechanical support to surrounding cells [12,13]. Furthermore, the artificially created ECM can modify cellular responses to soluble factors by binding, releasing, or activating signaling molecules [14]. For cellular growth, the scaffolds should be biocompatible, biodegradable, and with a suitable biodegradation rate to accommodate the neo-tissue regeneration rate. It is also desirable to have a scaffold with chemical composition and morphological structure similar to those of a native ECM, which can allow cell attachment, proliferation, and differentiation into a new tissue [15,16]. Natural polymers and synthetic biodegradable polymers such as gelatin [17], collagen [18], and poly(lactic acid–co-glycolic acid) [19] have been used extensively in tissue engineering with promising results. Synthetic polymers can provide good mechanical strength but poor biocompatibility. In contrast, natural polymers, as native ECM components, can promote cell attachment and proliferation but suffer from drawbacks like a fast degradation rate and low mechanical strength [20]. A feasible way to overcome these limitations is by blending two or more natural and synthetic polymers to fabricate a composite scaffold with improved properties [20,21]. Poly(caprolactone) (PCL) is a hydrophobic polymer with good mechanical strength and processing ability for many biomedical applications and has been approved by the Food and Drug Administration (FDA) for use in humans [22,23]. Nonetheless, with the inherent hydrophobicity and inertness of scaffolds made from PCL alone, an inferior cellular response is expected [24]. To improve this shortcoming, a composite scaffold can be prepared by blending PCL with a natural polymer, like gelatin, for tissue engineering purposes. As a collagen-derived material, the gelatin is easy to extract from tissues and has excellent cell adhesion, proliferation, and differentiation properties towards seeded cells [25]. Although a scaffold made from gelatin alone has low immunogenicity, its weak mechanical properties and high degradation rate demands incorporating PCL in the scaffold to drastically improve these deficiencies [26,27].

The electrospinning process is a widely employed technique for fabricating tissue engineering scaffolds [28]. This process can produce fibrous scaffolds under well-controlled conditions, like flow rates and concentrations of spinning solution, needle size, and the distance between the collector and needle tip. In addition, random and aligned fibers in micron to submicron scales can be prepared with natural and synthetic polymers using electrospinning [29]. In this way, an electrospun nanofiber membrane scaffold (NMS) with small pores but high porosity can allow nutrient diffusion and infiltration of seeded cells while preventing neighboring tissue penetration [30]. Undoubtedly, the main feature of an electrospun NMS is to mimic the complex three-dimensional structure of matrix fibers found in the ECM structure [31,32]. Therefore, electrospun NMSs have been widely used to regenerate different tissues, such as skin [33], tendon [34], bone [35], and cartilage [36], but few studies have been on regenerating mesothelium tissue [37]. Therefore, we prepare and characterize electrospun nanofibers from PCL and PCL/gelatin and use both NMSs for the in vitro culture of rat mesothelial cells. The best PCL/gelatin NMSs can successfully deliver autologous mesothelial cells to regenerate neo-mesothelium tissues in rats.

## 2. Results and Discussion

### 2.1. Characterization of PCL and PCL/Gelatin Nanofiber Membrane Scaffolds (NMSs)

**Figure 1:**
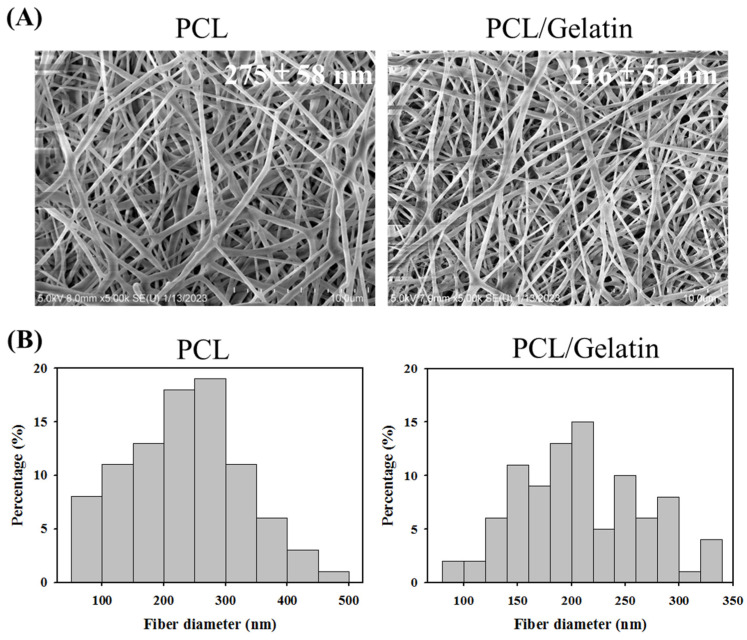
The scanning electron microscope (SEM) micrograph ((**A**), bar = 10 μm), and fiber diameter distribution (**B**) of PCL and PCL/gelatin nanofiber membrane scaffolds.

The scanning electron microscope (SEM) image of PCL NMSs delineates smooth fiber morphology devoid of beads with an average fiber diameter of 275 ± 58 nm (Figure 1A). By incorporating gelatin in the spinning solution, similar fiber morphology was observed for the PCL/gelatin NMSs. The fiber diameter shows some but insignificant change to 216 ± 52 nm. The distribution of the fiber diameter is uniform with 90% of fibers showing a fiber diameter of less than 300 nm for both NMSs (Figure 1B). The introduction of gelatin into the composite blend induces a slight reduction in fiber diameter, suggesting a possible interplay between the materials during the blending process to change the viscosity of the spinning solution [38,39]. The SEM images confirm the presence of a microporous structure within the membranes. This structural attribute implies a potential for nutrients and metabolic byproducts to permeate through the pores. However, the diminutive dimensions of these pores might pose challenges for cell infiltration and demand controlling the thickness of the fabricated membrane. In essence, the SEM characterization underscores the structural integrity and potential functionality of the electrospun membranes, laying a foundation for further investigation into their application for mesothelial cell culture.

**Figure 2:**
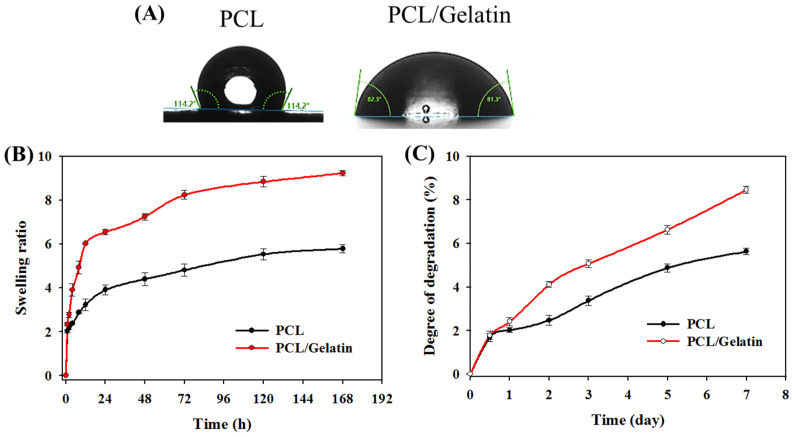
The water contact angle (**A**), swelling ratio (**B**), and degradation (in collagenase solutions) (**C**) of PCL and PCL/gelatin nanofiber membrane scaffolds.

To investigate the surface wettability of the scaffold, which plays an important role in cell attachment and proliferation, the water contact angle was determined to compare the difference in hydrophilicity of different NMSs (Figure 2A). The PCL NMS displays a water contact angle of 114.2°. By blending with gelatin, the water contact angle of the PCL/gelatin NMS drastically reduced by 28% to 82.3° to increase surface wettability. The increase in wettability for the PCL/gelatin NMS is mediated by the hydrophilic nature of gelatin, which is a highly hydrophilic protein material with excellent biocompatibility and biodegradability [40]. By reducing the water contact angle after blending with PCL, the hydrophilic surface of PCL/gelatin nanofibers is expected to influence the attachment and proliferation of mesothelial cells.

The swelling ratio of the NMSs shown in Figure 2B indicates very fast water absorption kinetics. The swelling ratio reaches ~5 or ~9 in 120 h, depending on the type of NMS. The swelling ratio of PCL/gelatin is higher than that of PCL due to its higher hydrophilicity, which supports the results of the contact angle measurements. The degradability of PCL and PCL/gelatin NMSs in collagenase solution at different time intervals was evaluated. Unlike, PCL, gelatin as a denatured protein from collagen can be degraded by collagenase, which makes PCL/gelatin NMSs degrade faster than PCL/gelatin NMSs (Figure 2C) [41]. Collagenase can only break down gelatin but not the main component PCL in the PCL/gelatin nanofibers. It can hydrolyze peptide bonds in the Pro-X-Gly-Pro repeated amino acid sequence in gelatin through proteolytic cleavage of a peptide bond formed between glycine (Gly) and a neutral amino acid (X). With the accelerated enzymatic degradation study in a collagenase solution, the degradation study underlines a faster deterioration rate of PCL/gelatin NMSs in vivo. Nonetheless, improved membrane hydrophilicity may be another possible cause of the enhanced degradation rate observed for PCL/gelatin NMSs in collagenase solution.

**Figure 3:**
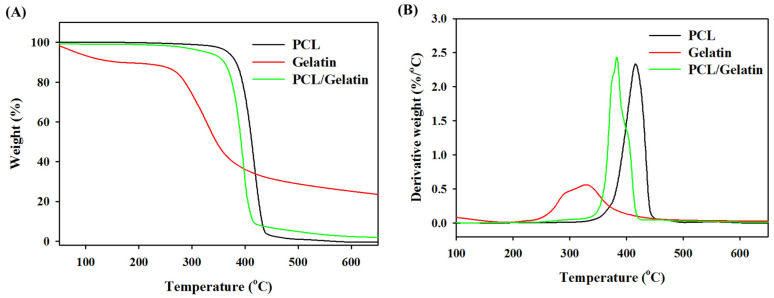
The thermogravimetric analysis (TGA) (**A**) and the differential thermal gravimetric (DTG) analysis (**B**) of the PCL nanofiber membrane scaffold, gelatin, and PCL/gelatin nanofiber membrane scaffold.

The thermal stability of PCL NMSs, PCL/gelatin NMSs, and gelatin powder were studied by TGA and DTA in Figure 3A,B. The TGA is a technique that measures the mass of a material as it is heated up while the DTA can measure the difference in temperature between the sample and a reference material as they are both heated up. These techniques can be used to study the thermal stability of materials and to identify the temperature at which they decompose. The PCL NMS shows single-staged thermal decomposition starting from 346 °C and ending at 430 °C. A peak temperature at 419 °C was found for PCL, and there was only 0.5% residual weight after complete decomposition at 650 °C. Gelatin shows some weight loss within 50 and 120 °C due to evaporation of absorbed water. A second-stage weight loss for gelatin is noted between 250 and 420 °C, corresponding to the thermal degradation of this protein. The peak temperature is 330 °C for gelatin, and the residual weight is 24% due to its natural polymer nature [42]. The PCL/gelatin NMS depicts thermal degradation starting at 275 °C and completing at 420 °C, leading to 2.0% residual weight due to the gelatin in the blend. As the calculated weight percent of gelatin in the PCL/gelatin NMS is 9.1%, the theoretical residual weight of this scaffold is 2.2% and close to the value obtained from TGA. There are two decomposition peak temperatures observed from the DTA curve: the first small broad peak starting as early as ~318 °C corresponded to gelatin degradation, and the second one corresponded to PCL decomposition between 383 and 407 °C [43]. The interaction between gelatin and PCL in the PCL/gelatin NMS may shift the peak temperature assigned to PCL in the PCL NMS (419 °C) to a lower value.

**Figure 4:**
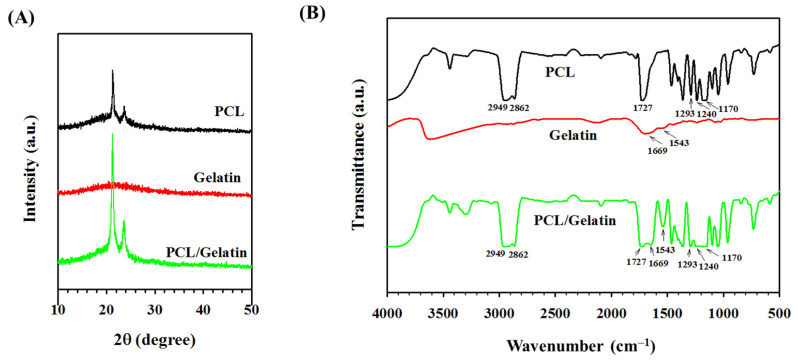
The X-ray diffraction (XRD) (**A**), and Fourier-transform infrared (FTIR) spectroscopy (**B**) analysis of the PCL nanofiber membrane scaffold, gelatin, and PCL/gelatin nanofiber membrane scaffold.

Figure 4A shows the X-ray diffraction (XRD) analysis results of PCL and PCL/gelatin NMSs, as well as gelatin powder. The PCL NMS shows a sharp crystalline peak at 21.4° (110) and a less sharp semi-crystalline peak at 23.7° (200). In contrast, as a protein material, gelatin shows a broad peak at 22.9°, indicating its amorphous nature. The PCL/gelatin NMS shows the two characteristic peaks of PCL at 21.9° and 23.7°, indicating gelatin will not change the degree of crystallinity of PCL/gelatin from the PCL NMS by completely dissolving both components in the solvent (formic acid) to prepare a well-mixed spinning solution without phase separation [26].

Figure 4B represents the Fourier-transform infrared (FTIR) spectra of the PCL NMS, PCL/gelatin NMS, and gelatin powder. Several PCL characteristic bands were observed at 1170 cm^−1^ (symmetric C-O-C stretching), 1240 cm^−1^ (asymmetric C-O-C stretching), 1293 cm^−1^ (C-O and C-C stretching), 1727 cm^−1^ (carbonyl stretching), 2862 cm^−1^ (symmetric CH_2_ stretching), and 2949 cm^−1^ (asymmetric CH_2_ stretching) [26,44]. The gelatin showed bands at amide II bending at 1543 cm^−1^ and C=O stretching at 1669 cm^−1^ [42]. In the PCL/gelatin NMS, all characteristic peaks corresponding to PCL and gelatin were observed, suggesting all components were successfully integrated into the PCL/gelatin NMS prepared by electrospinning a blend polymer solution.

**Figure 5:**
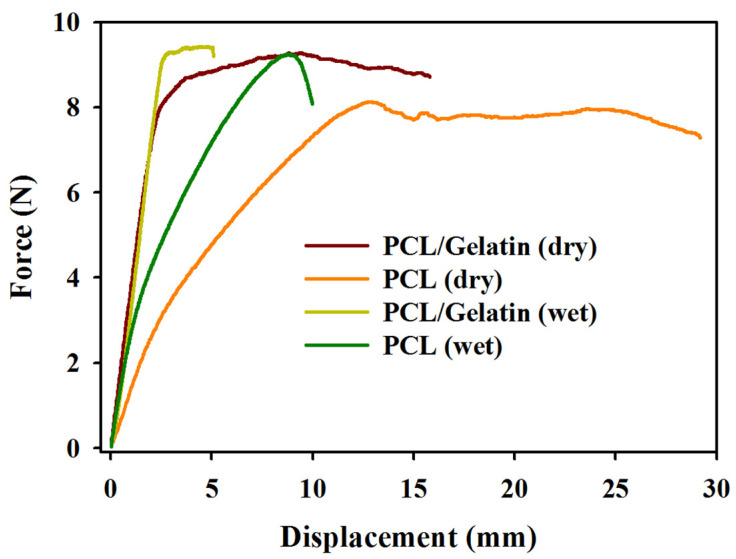
Typical tensile force–displacement curves of PCL and PCL/gelatin nanofiber membrane scaffolds in dry and wet conditions.

An NMS should have sufficient strength to resist the stress experienced during application and neo-tissue regeneration. For this purpose, a uniaxial tensile test was conducted in a mechanical testing machine to determine material parameters such as stiffness, ultimate strength, and elongation at break of dry and wet NMSs. The wet scaffold was prepared by immersing in PBS for 7 days at 37 °C to simulate the cell culture condition. Figure 5 shows typical force vs. displacement curves of the PCL and PCL/gelatin NMSs. The average values of stiffness, maximum load, and maximum displacement were calculated from four samples (*n* = 4) and are included in Table 1. A macroporous membrane composed of polymers will exhibit an initial elastic region showing a linear line in a force vs. displacement curve. This line bends before breakage. In dry conditions, the PCL/gelatin NMS displays comparable maximum load and lower maximum displacement but higher stiffness than PCL, as shown in previous reports [45,46]. Similar trends in mechanical parameters difference between PCL and PCL/gelatin for both dry and wet samples indicate the NMSs can maintain their mechanical properties during in vitro cell culture and in vivo implantation. The presence of a crystalline domain, which makes gelatin flexible, may be responsible for the slight increase in maximum load upon mixing with gelatin.

### 2.2. In Vitro Study

**Figure 6:**
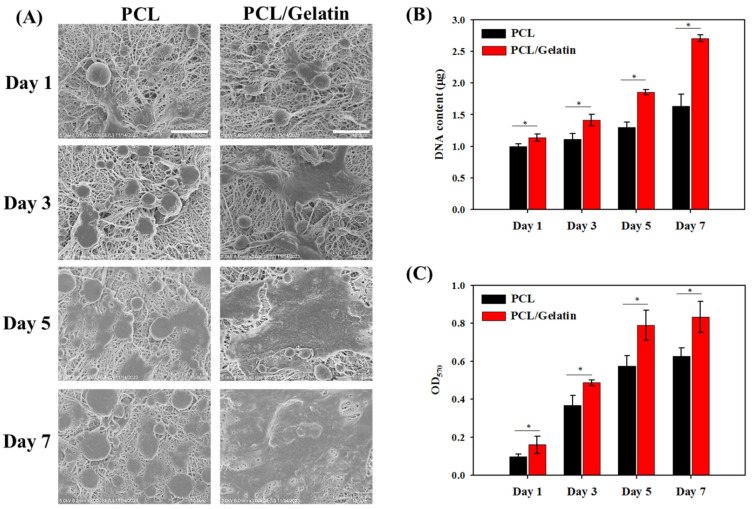
(**A**) The scanning electron microscopy (SEM) analysis of mesothelial cells in PCL and PCL/gelatin nanofiber membrane scaffolds (bar = 10 μm). The proliferation of mesothelial cells in PCL and PCL/gelatin nanofiber membrane scaffolds from cellular DNA contents (**B**) and MTT assays (**C**). * *p* < 0.05.

The morphology of attached mesothelial cells in the PCL and PCL/gelatin NMSs after different culture times was examined using an SEM (Figure 6A). After cell seeding on day 1, the mesothelial cells adhered smoothly to the nanofiber surface and began to spread, revealing spindle cell morphology. By day 3, the cells exhibited a polygonal shape, resembling the characteristic cobblestone pattern of mesothelial cells. There were more cells in PCL/gelatin compared to the PCL. Throughout days 5 and 7, a thin cellular coating developed on the scaffold’s surface. The cells maintained their epithelial-like characteristics during growth, although they were more elongated and flattened. A continuous cell layer with a higher cell population was noted on the surface of the PCL/gelatin NMS. In general, the SEM analysis confirms the preservation of the mesothelial phenotype of attached mesothelial cells in NMSs. A layer of ECM appears to cover the strongly adhered cells.

To evaluate cell attachment and proliferation, the cell number in the PCL and PCL/gelatin NMSs was compared by determining the total DNA contents in each scaffold (Figure 6B). A noticeable increase in DNA content was observed with time. The DNA content on day 1 is significantly higher for PCL/gelatin than PCL, indicating that gelatin can promote cell attachment. Additionally, an interesting observation was made regarding the impact of gelatin. Even though the DNA content increased in both scaffolds, it is worth noting that PCL/gelatin may offer a more favorable environment for mesothelial cell growth. Indeed, the DNA content increase rate from day 1 to day 7 is significantly higher for PCL/gelatin (2.7 folds) than for PCL (1.6 folds), which can be attributed to a higher cell proliferation rate induced by gelatin. Gelatin has a repeated Arg-Gly-Asp (RGD) tripeptide sequence in its amino acid sequence to function as a cell-recognition motif. This RGD sequence can interact with integrin, a receptor family for ECM proteins on the cell surface to mediate cell adhesion and promote cell attachment [47]. Furthermore, the integrin can function as a signal transducer, which can activate various intracellular signaling pathways when activated by binding RGD, and the cooperation between RGD and integrin may promote cell proliferation [48]. For tissue engineering applications, incorporating gelatin in the scaffold can significantly enhance cell attachment to the scaffold through the RGD–integrin interactions, which can also activate intracellular signaling pathways to upregulate gene expression related to cell growth and differentiation [49]. The cell proliferation rate was also studied by measuring the metabolic activity of mesothelial cells in the NMSs by MTT assays. As shown in Figure 6C, the increase in viable cell number follows a similar trend as observed from the DNA content, endorsing the biocompatibility of NMSs for cell proliferation.

**Figure 7:**
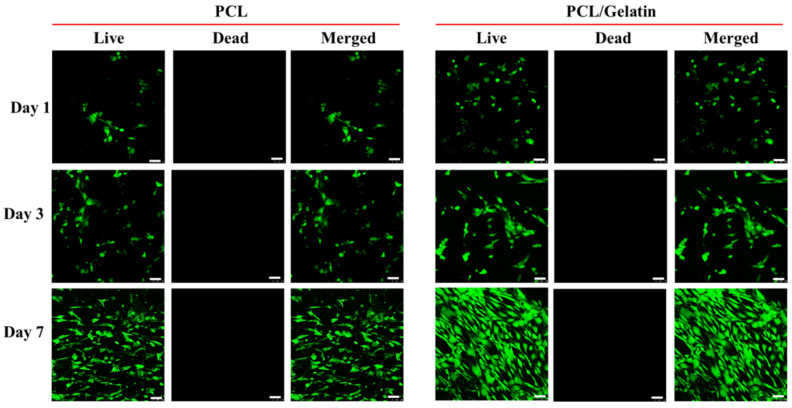
The confocal microscopy images after live/dead staining of mesothelial cells in PCL and PCL/gelatin nanofiber membrane scaffolds. Bar = 50 μm.

The cell viability was evaluated by live/dead staining and examined by laser scanning confocal microscopy. As shown in Figure 7, stacked confocal images indicate that the seeded cells are highly viable throughout the observation period from day 1 with negligible dead cells. The PCL/gelatin exhibits more live cells than PCL with elongated cell morphology on day 3. More sporadically distributed live cells showing elongated shapes were found on day 3 than on day 1 and still no dead cells could be observed as before. On day 7, gelatin-mediated cell proliferation leads to the vast increase in mesothelial cells in PCL/gelatin with an increased cell population distributed evenly within the scaffold. Additionally, a single layer of mesothelial cells developed in the NMSs may be sufficient to maintain the mesothelial phenotype and cell viability since only a few layers of mesothelial cells exist in a mesothelium layer in vivo. The macroporous nature of PCL and PCL/gelatin NMSs is anticipated to enable sufficient gas and nutrition exchange to maintain high cell viability and promote cellular growth for engineering a mesothelium layer.

**Figure 8:**
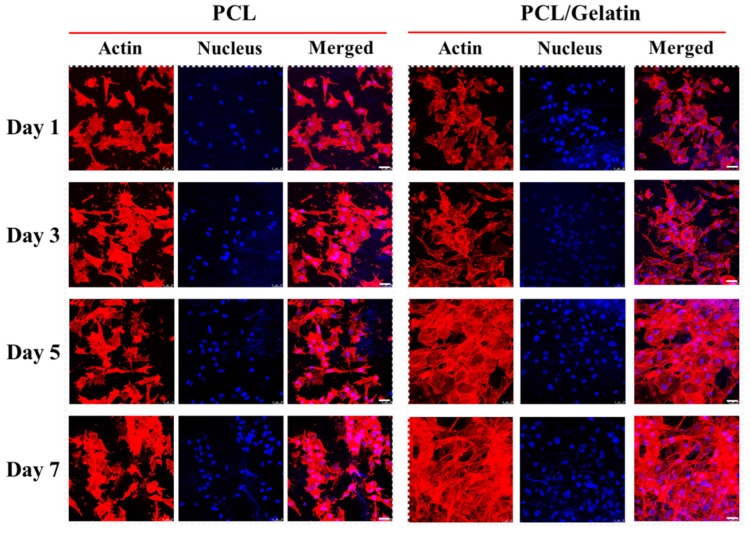
Confocal microscopy images after actin cytoskeleton and nucleus staining of mesothelial cells in PCL and PCL/gelatin nanofiber membrane scaffolds. Bar = 25 μm.

The mesothelial cell phenotypes were observed by staining the cell cytoskeleton and nucleus and imaged by confocal microscopy (Figure 8). A spindle-shaped morphology was observed for mesothelial cells with an intact cytoplasmic filamentous distribution of F-actin (red) and oval nuclei (blue). The results of the cytoskeleton staining agree well with the SEM observation at all time points, where scattered cells were found attached well to the nanofibers. Notably, for the mesothelial cells seeded onto PCL/gelatin, increased alignments of the cells were observed on days 5 and 7 post-seeding. On day 7, the PCL/gelatin NMSs showed a confluent layer of cells spread throughout the membrane scaffolds. Overall, these results illustrated that the PCL/gelatin NMSs could increase the attachment and proliferation of mesothelial cells, endorsing its preference for use in mesothelial cell delivery.

**Figure 9:**
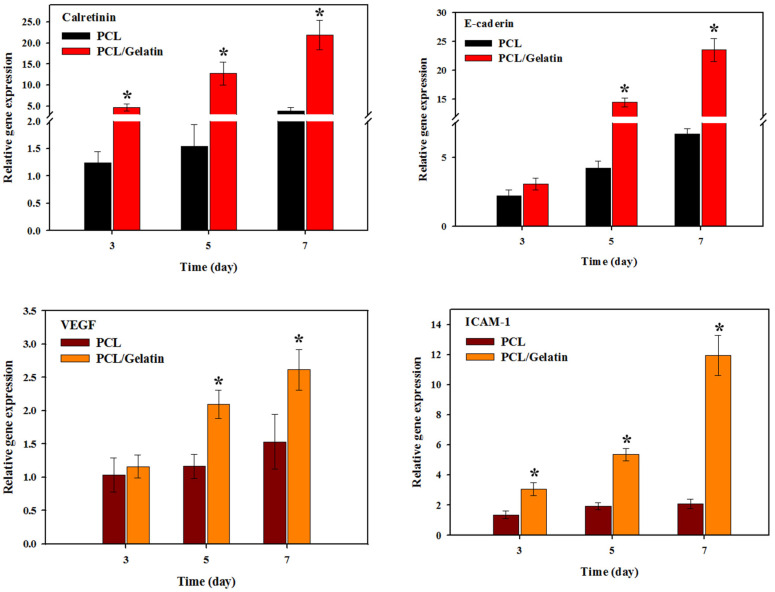
Quantitative real-time polymerase chain reaction (qRT-PCR) for quantifying the relative (normalized to day 1) gene expression levels of calretinin, E-cadherin, vascular endothelial factor (VEGF), and intercellular adhesion molecule (ICAM-1) by mesothelial cells in PCL and PCL/gelatin nanofiber membrane scaffolds. The glyceraldehyde 3-phosphate dehydrogenase (GAPDH) was used as a control. * *p* < 0.05 compared with PCL.

The expression of mesothelial cell marker genes, including calretinin, E-cadherin, vascular endothelial factor (VEGF), and intercellular adhesion molecule (ICAM-1), were quantified using quantitative real-time polymerase chain reaction (qRT-PCR) (Figure 9). Mesothelial cells secrete calretinin as a calcium-binding protein, which is involved in calcium signaling. Although the precise role of calretinin remains little elucidated, there is a hypothesis that it influences the cell cycle [50]. However, several immunohistochemistry investigations have shown that calretinin could be a valuable marker protein for mesothelial cells [51,52]. Mesoderm is the source of mesothelial cells, which are distinct in that they express many cell adhesion molecules, such as ICAM-1 and E-cadherin. E-cadherin is a calcium-dependent transmembrane epithelial adhesion protein uniquely expressed in epithelial lineage cells and facilitates intercellular adhesion [53]. Acquiring mesenchymal migratory characteristics for epithelial cells is related to the loss of E-cadherin expression, indicating this protein plays a critical role in overseeing the epithelial-to-mesenchymal transition [54]. The ICAM-1 or CD54 is localized in the plasma membrane and cytoplasm, and mesothelial cells constitutively express ICAM-1. It has been shown that ICAM-1 has promising potential as a distinguishing factor between fibroblasts and mesothelial cells. The introduction of soluble ICAM-1 into cells leads to a significant decrease in neutrophil transmigration [55,56]. When the mesothelial cells were cultured in the PCL/gelatin NMS, the mesothelium marker genes were upregulated compared to PCL. This supports the idea that gelatin can help mesothelial cells keep their phenotypic properties during cell division and growth. As a potent proangiogenic factor, the VEGF is involved in the proliferation of endothelial cells and permeability of the vasculature [57]. This protein is an important growth factor for mesothelial cells to stimulate their growth. Transient production of VEGF by mesothelial cells appears to play an important role in the angiogenesis of peritonea. It is known that epithelial cells tend to lose their cell–cell adhesion and cell polarity by gaining migratory and invasive properties during the epithelial–mesenchymal transition process, and this process is regulated by VEGF [58]. Transitional local production of VEGF by mesothelial cells in the presence of gelatin in the PCL/gelatin NMS appears to play a crucial role in maintaining the characteristics of mesothelial cells in this scaffold [59].

**Figure 10:**
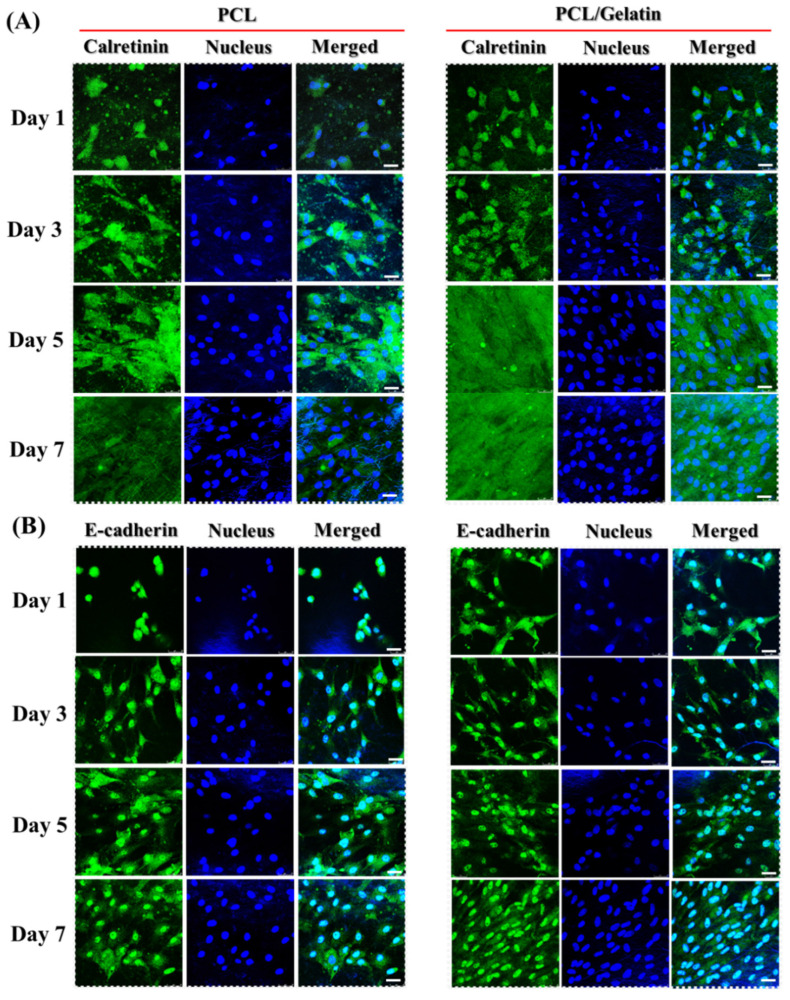
The immunofluorescence staining of calretinin (**A**) and E-cadherin (**B**) via labeling with fluorescein-conjugated antibody after culturing mesothelial cells in PCL or PCL/gelatin nanofiber membrane scaffolds. The nucleus was stained with Hoechst 33258 to show blue fluorescence. Bar = 25 μm.

The expression of mesothelial marker proteins (calretinin and E-cadherin) was studied by immunofluorescence (IF) staining under a confocal microscope (Figure 10A,B). The seeded mesothelial cells could synthesize both proteins on day 1 as the cells proliferated in the scaffold. There was a consistent and ongoing increased expression of calretinin and E-cadherin from day 3 to day 7, as evidenced by the time-lapsed accumulation and distribution of these proteins in the scaffolds. However, the PCL/gelatin appears to have a higher rate of synthesis in the mesothelial marker proteins, especially in the case of calretinin. To compare the scaffold-dependent protein expression, we performed a semi-quantitative analysis of the area of the green fluorescence-stained regions in the confocal images. The synthesis of calretinin and E-cadherin differs significantly between scaffolds at each time point as anticipated (Table 2), suggesting that these essential mesothelial ECM proteins are efficiently synthesized in conjunction with cell proliferation. The incorporation of gelatin into PCL/gelatin NMSs significantly enhanced the synthesis of calretinin and E-cadherin and supports the use of this scaffold to offer an ECM-like milieu to preserve the mesothelial phenotype for mesothelial cell delivery to repair damaged mesothelium.

### 2.3. In Vivo Study

**Figure 11:**
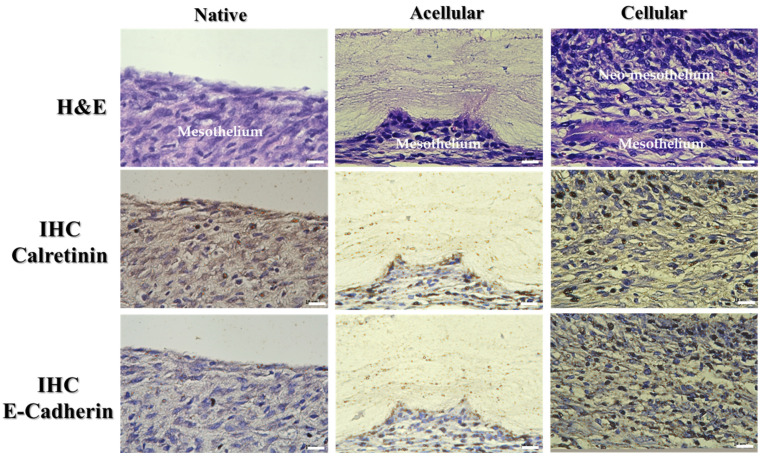
The hematoxylin and eosin (H&E) and calretinin and E-cadherin immunohistochemical (IHC) staining of retrieved mesothelium tissue 7 days post-implantation (bar = 10 μm). The abraded parietal peritoneum was repaired with PCL/gelatin nanofiber membrane scaffolds (NMSs) or PCL/gelatin NMSs + mesothelial cells as the acellular and the cellular group, respectively. The PCL/gelatin + mesothelial cell construct is prepared by culturing mesothelial cells in a PCL/gelatin NMS for 7 days in vitro before implantation. The native mesothelium tissue is shown on the left for comparison.

Using PCL/gelatin NMSs, we used scaffold-only (acellular groups) or the cell/scaffold construct (in vitro culture for 7 days) to repair damaged mesothelium in rats in an allograft cell transplantation study. The peritoneum tissue specimens were retrieved from the animals and examined histologically 7 days post-implantation. A histological analysis of the repaired mesothelium was conducted 7 days post-implantation. The hematoxylin and eosin (H&E) staining showed typical mesothelial cells with flat and elongated morphology on the surface of the native mesothelium and the repair mesothelium only in the cellular group as expected (Figure 11). When the cell/construct covers the damaged mesothelium, a neo-mesothelium layer closely resembling native mesothelium forms on top of the undamaged mesothelium although with a loose structure at the junction of new and native tissue layers. However, the wound covered with a PCL/gelatin NMS in the acellular group does not reveal any mesothelium regeneration in the upper layer of the damaged mesothelium. Indeed, the top layer of the retrieved mesothelium in the cellular group features a layer of mesothelial cells forming a mesothelium layer closely resembling the native mesothelium in as short as 7 days. This indicates that PCL/gelatin NMSs can provide a suitable milieu for delivering mesothelial cells. The H&E staining image also revealed some residual membrane filaments in the acellular group, adhering to the wound with a light purple color.

The stained brown color around the cell nuclei in the cellular group identified the key mesothelial marker proteins produced by transplanted mesothelial cells from immunohistochemical (IHC) staining of calretinin and E-cadherin for indicating the continued production of mesothelial marker proteins by mesothelial cells in the transplanted allograft to help fix the damaged mesothelium. In H&E staining, the formation of a neo-mesothelium layer like a native mesothelium was noted in the cellular group, where abundant key mesothelial cell marker proteins calretinin and E-cadherin were produced by the transplanted mesothelial cells. The uppermost layer of the parietal peritoneum surface is where mesothelial cells should be found to perform their normal physiological functions. A loose structure at the interface of the native and new mesothelium layer in the cellular group may demand a longer implantation time. The neo-mesothelium tissue produced is expected to be re-modeled, and a functional mesothelium will be developed from the allograft mesothelial cells with NMS-mediated cell transplantation to repair damaged mesothelium. Overall, a neo-mesothelium tissue similar to the native mesothelium tissue was noted from the cellular group from both H&E and IHC staining.

## 3. Materials and Methods

### 3.1. Materials

Gelatin from porcine skin (Type A), poly (ε-caprolactone) (PCL, molecular weight = 80,000 Da), and Dulbecco’s modified Eagle’s medium (DMEM), fetal bovine serum (FBS), penicillin/streptomycin solution, and trypsin-EDTA were acquired from Sigma-Aldrich (St. Louis, MO, USA). Hoechst 33258, Phalloidin tetramethylrhodamine B isothiocyanate (Phalloidin-TRITC), and a live/dead viability/cytotoxicity kit were purchased from Thermo Fisher Scientific (Waltham, MA, USA).

### 3.2. Preparation of Electrospun Nanofiber Membrane Scaffolds (NMSs)

The PCL NMS was prepared from a 15% (*w*/*v*) PCL solution prepared in formic acid. The PCL/gelatin NMS was prepared from 15% (*w*/*v*) PCL and 1.5% (*w*/*v*) gelatin solution prepared in formic acid. A 10 mL syringe was mounted on a syringe pump (KD Scientific Co., Holliston, MA, USA) operated at 0.5 mL/h and fitted with a 21-gauge blunt needle. The polymer solution was horizontally drawn from the needle tip and collected with an aluminum foil-covered collector, which was placed 15 cm from the needle tip. The temperature was at room temperature, and the relative humidity was controlled at ~64%. A high-voltage power supply operated at 23 kV provided an electrostatic force between the grounded collector and the needle tip to eject the polymer solution, and PCL and PCL/gelatin NMSs with ~300 μm thickness were collected with the collector.

### 3.3. Characterization of Electrospun Nanofiber Membrane Scaffolds (NMSs)

The morphology of the electrospun nanofibers was examined using a JEOL JSM-7500F scanning electron microscope (SEM). To determine the average fiber diameter from the SEM images, ~20 nanofibers were randomly chosen from each SEM image, and 5 SEM images were used to calculate the fiber diameter using ImageJ software (ImageJ 1.53j java 1.8.0_112 (64-bit) version). To measure the water contact angle, the sessile-drop method was employed, using an FTA-125 contact angle/surface tension machine from First Ten Angstroms (Portsmouth, VA, USA). After dropping 2 μL of distilled water on the NMSs, the contact angle was calculated with DinoCapture 2.0 software.

The swelling ratio of the NMSs was studied using the gravimetric method. The NMSs were dried at 50 °C to a constant weight, and the dry weight was measured (*W_d_*). After immersing in distilled deionized (DDI) water at room temperature for different times, the NMSs were retrieved from the solution and excess water on membrane surface was removed with a filter paper. The wet weight of the NMSs was measured (*W_w_*). The swelling ratio was calculated from $\left(W_{w}-W_{d}\right)÷W_{d}$ (*n* = 4).

For the degradation study of NMSs in a collagenase solution at 37 °C, the dry weight (*W*_1_) of the NMSs was measured. The pre-weighed NMSs were placed in a 1.5 mL tube and incubated statically with 1 mL enzyme solution containing 2 U of collagenase. After retrieving the samples at different times, the NMSs were washed three times with DDI water and dried to a constant weight (*W*_2_) at 50 °C. The degree of degradation (%) was calculated from $\left(W_{1}-W_{2}\right)÷W_{1}\times 100$ (*n* = 4).

The thermogravimetric analysis (TGA) was conducted with TGA Q50 from TA Instruments (New Castle, DE, USA). The NMSs (5 to 10 mg) were cut into pieces and placed on a standard aluminum pan. The analysis was carried out by heating the pieces to 700 °C in nitrogen at 10 °C/min. An X-ray diffraction (XRD) analysis was conducted with a Bruker D2 Phaser X-ray diffractometer (Billerica, MA, USA) with a Cu Kα X-ray source (λ = 1.5406 Å) with a 2θ angle from 10 to 50°. An attenuated total reflectance–Fourier-transform infrared (ATR-FTIR) spectroscopy analysis was conducted with a Bruker Tensor 27 FTIR spectrophotometer (Billerica, MA, USA) from 500 to 4000 cm^−1^.

The tensile mechanical properties of the NMSs were evaluated using a Tinius Olsen H1KT mechanical testing machine (Horsham, PA, USA) using a 100 N load cell. Other than the as-prepared dry NMSs, wet scaffolds immersed in PBS for 7 days at 37 °C were also tested. By uniaxially loading a 50 mm (length) × 10 mm (width) membrane at a deformation rate of 5 mm/min, a force–displacement curve could be obtained. The maximum loading force, maximum displacement, and stiffness (slope of the force–displacement curve in the initial linear region) were determined for 4 samples.

### 3.4. In Vitro Cell Culture

To isolate mesothelial cells from Sprague Dawley (SD) rats, the skin of the lower abdomen was sterilized and cut with a blade. The peritoneum was cut into 2 × 2 cm pieces and washed extensively with phosphate-buffered saline (PBS). The peritoneum tissue was digested in a collagenase solution (0.2%) and filtered with a 70 μm pore size filter to remove cell debris. After neutralizing the enzyme activity with cell culture medium (90% DMEM and 10% FBS), a cell pellet was obtained by centrifugation at 200× *g* for 3 min. After re-suspending in the cell culture medium, a cell suspension was obtained for seeding on a disc-shaped NMS (12 mm diameter). The NMS was sterilized with 75% ethanol, rinsed twice with PBS, and placed in a 24-well culture plate for cell seeding. An aliquot of 10 μL cell suspension containing 1 × 10^5^ cells was seeded onto the surface of the membrane and incubated at 37 °C for 4 h. After cell adhesion, the membrane was removed and placed into a new well and cultured with 1 mL culture medium at 37 °C in a CO_2_ incubator. The cell culture medium was changed every two to three days.

### 3.5. Cell Morphology by Scanning Electron Microscope (SEM) Observation

The cell-seeded scaffold was removed from the cell culture plate on days 1, 3, 5, and 7, washed 3 times in PBS, and fixed with 10% formaldehyde. After dehydrating with gradient ethanol from 50% to 99.5%, the samples were air-dried and sputter-coated with gold. A Hitachi S3000N scanning electron microscope (SEM) (Tokyo, Japan) was used to examine cell morphology from the membrane surface.

### 3.6. Cell Proliferation by DNA Contents and MTT Assays

A cell-seeded NMS was removed from the culture plate and digested in a cell lysis solution containing 55 mM sodium citrate, 150 mM sodium chloride, 5 mM cysteine, 5 mM EDTA, and 0.2 mg/mL papain for 24 h at 60 °C. After centrifugation, the DNA content in the supernatant was quantified using Hoechst 33258 at 360 nm excitation wavelength and 460 nm emission wavelengths using a standard curve constructed from calf thymus DNA. For the MTT assays, the cell culture medium was removed, and 0.1 mL of 3-(4,5-Dimethylthiazol-2-yl)-2,5-diphenyltetrazolium bromide (MTT) solution (0.5 mg/mL) was added for incubation at 37 °C for 3 h. After removing the MTT solution, 0.1 mL of dimethyl sulfoxide was used to fully dissolve the formed formazan purple crystal by gently shaking the plate to obtain a uniform purple color. The solution absorbance (optical density, OD) was determined from a plate reader at 570 nm (OD_570_).

### 3.7. Quantitative Real-Time Polymerase Chain Reaction (qRT-PCR)

The gene expression of calretinin, E-cadherin, VEGF, and ICAM-1 was analyzed by qRT-PCR. After isolating total RNA using TRIzol (Invitrogen, Carlsbad, CA, USA), the complementary DNA (cDNA) was obtained using a Maxime RT premix kit (Intron Biotechnology, Seoul, Republic of Korea) according to standard protocols, and glyceraldehyde-3-phosphate dehydrogenase (GAPDH) was used as a housekeeping control. For real-time PCR reactions, amplification was conducted for 45 cycles, 10 min at 95 °C for denaturation, 30 s at 95 °C for annealing, and 1 min at 67.1 °C (E-cadherin, GAPDH, ICAM-1 and VEGF) and 60.9 °C (calretinin) for extension. An SYBR Green RT-PCR kit (SYBR Green I SuperMix, Bio-Red Laboratories Inc., Hercules, CA, USA) was used to visualize the PCR products in real-time. The primers were supplied by Tri-I Biotech Inc. (Taipei, Taiwan) with the sequence shown below. GAPDH: forward 5′CACCATCTTCCAGGAGCGAG3′, reverse 5′GGCGGAGATGATGACCCTTT3′. E-cadherin: forward 5′AAGGGCTTGGATTTTGAGG3′, reverse 5′AGATGGGGGCTTCATTCAC3′. Calretinin: forward 5′TATCCAGCAGCTCACCACCTAC3′, reverse 5′GAGAGGTCTGGGAAGGAGTTTC3′. ICAM-1: forward 5′GCCTGGGGTTGGAGACTAAC3′, reverse 5′CTGTCTTCCCCAATGTCGCT3′. VEGF: forward 5′TGAGACCCTGGTGGACATCT3′, reverse 5′CTCCTATGTGCTGGCTTTGG3′. The relative gene expression was reported by normalizing the gene expression levels on day 3, 5, and 7 with that on day 1.

### 3.8. Cell Viability by Live/Dead Staining

The cell viability was determined using a live/dead viability/cytotoxicity kit containing Calcein AM and propidium iodide. After washing the cell-seeded NMS with PBS and staining for 15 min at 37 °C, the sample was imaged under a Leica TCS SP2 laser scanning confocal microscope (Wetzlar, Germany). The live cells showed green fluorescence at 494/517 nm excitation/emission wavelengths, and the dead cells showed red fluorescence at 528/617 nm excitation/emission wavelengths.

### 3.9. Cytoskeleton Staining

The cytoskeleton expression of adhered mesothelial cells in the NMSs was assessed from F-actin staining by staining with Phalloidin-TRITC. The cell-seeded membranes were fixed in 10% formaldehyde, washed with PBS, and permeabilized with 1% Triton X-100 for 5 min. The F-actin was stained with 50 μg/mL Phalloidin-TRITC for 30 min, and the cell nuclei were stained with 10 μg/mL Hoechst 33258 for 10 min. The cytoskeletal arrangement was observed with a Leica TCS SP8 laser scanning confocal microscope (Wetzlar, Germany) at 340 nm/488 nm excitation/emission wavelengths for cell nuclei and 540 nm/570 nm excitation/emission wavelengths for cytoskeleton expression.

### 3.10. Immunofluorescence (IF) Staining

To elucidate the protein expression of calretinin and E-cadherin of seeded mesothelial cells, immunofluorescence (IF) staining was conducted by fixing the sample with 10% formaldehyde at 4 °C for 60 min. The fixed sample was washed with PBS containing 1% Tween 20 (PBST) and blocked with a Hyblock blocking buffer. The sample was incubated with rabbit anti-mouse primary antibody against calretinin or E-cadherin at 4 °C overnight. After rinsing in PBST, the sample was incubated with a FITC-conjugated AffiniPure goat anti-rabbit IgG secondary antibody at 37 °C for 2 h. After another PBST washing, Hoechst 33258 was used to label the cell nuclei. The sample was imaged under a Leica TCS SP8 laser scanning confocal microscope (Wetzlar, Germany) where calretinin and E-cadherin were observed at an excitation/emission wavelength of 490 nm/525 nm for fluorescein and Hoechst 33258 at an excitation/emission wavelength of 350 nm/461 nm. A semi-quantitative protein expression analysis from IF staining was carried out using ImageJ software (ImageJ 1.53j java 1.8.0_112 (64-bit) version) by measuring the green fluorescence area in each image (*n* = 4).

### 3.11. In Vivo Studies

Male SD rats were used for this study following protocols approved by the Institutional Animal Care and Use Committee of Chang Gung University (IACUC approval no. CGU111-237). With isoflurane-induced general anesthesia, the rats were randomly divided into two groups (acellular and cellular) for surgery. The depilated lower abdomen was cleaned with betadine and alcohol, and a 5 cm diameter C-shaped incision was made sterilely for laparotomy. To create a 15 mm diameter wound area, a sterilized toothbrush was used to abrade the parietal peritoneum with 100 strokes, after which a punctate bleeding surface was obtained. The wound in the acellular group was covered with a 2 cm diameter PCL/gelatin NMS pre-immersed in PBS at 37 °C for 7 days. The wound in the cellular group was covered with a 2 cm diameter cell-seeded PCL/gelatin NMS, which was prepared with 3 × 10^5^ mesothelial cells and cultured at 37 °C for 7 days in vitro. After treatment, the abdominal wall was closed with a 3-0 nylon running suture, and the skin was closed with a 3-0 nylon interrupted suture. On day 7 post-implantation, the animals were euthanized with CO_2_, and the peritoneum tissue specimen was retrieved and fixed in 10% formaldehyde. After embedding in paraffin and sectioning in 5 µm thickness, the specimen was subject to histological evaluation with hematoxylin and eosin (H&E) staining. For immunohistochemical (IHC) staining, the specimen was deparaffinized and washed three times in PBST for 5 min. Hydrogen peroxide treatment (10 min) was used to block non-specific binding, and the specimen was washed with PBST for 15 min. The sections were incubated for 60 min at room temperature in a rabbit anti-calretinin polyclonal primary antibody solution or a rabbit anti-E-cadherin polyclonal primary antibody solution in a humid environment. The slides were washed in PBST for 5 min, and HRP Polymer Quanto was applied for 10 min and washed for 5 min. The slide was incubated with a 3-diaminobenzidine (DAB) solution for 1 min for color development, counterstained with hematoxylin for 30s, and observed under an inverted optical microscope.

### 3.12. Statistical Analysis

All of the data were expressed as the mean ± standard deviation (SD). A one-way analysis of variance (ANOVA) was used for the statistical analysis with the least significant difference (LSD) test, with *p* < 0.05 considered a significant difference.

## 4. Conclusions

By blending PCL and gelatin in a spinning solution, a composite PCL/gelatin NMS can be fabricated with increased hydrophilicity and improved mechanical properties while providing an improved milieu for mesothelial cell culture. The addition of gelatin shows a significant impact on the cellular response of attached mesothelial cells, without changing fiber diameters. The in vitro cell culture experiments show that mesothelial cells grow well in the NMSs and show high cell viability. The mesothelial cells displayed a higher cell proliferation rate, better maintenance of cell morphology, and enhanced phenotypic expression in the PCL/gelatin NMSs, as indicated by the upregulated gene and protein expression levels of calretinin and E-cadherin, which are marker proteins crucial for determining mesothelial identity. The animal studies demonstrated that the PCL/gelatin NMS is a potential delivery vehicle for allografting mesothelial cells onto damaged mesothelia in rat abrasion models. By using PCL/gelatin NMSs for the delivery of allograft mesothelial cells, the cell/scaffold construct can heal the injured mesothelium tissue in the lower abdomens of rats. An implantation of a cell-seeded PCL/gelatin NMS can provide a cell delivery method to an injured mesothelium site to facilitate the generation of neo-mesothelium tissues from transplanted mesothelial cells.

## Figures and Tables

**Table 1:** The tensile mechanical properties of PCL and PCL/gelatin nanofiber membrane scaffolds.

Scaffolds	Maximum Load (N)	Maximum Displacement (mm)	Stiffness (N/mm)
PCL (dry)	8.56 ± 0.95	25.00 ± 3.63	1.26 ± 0.06
PCL/gelatin (dry)	9.19 ± 0.41	14.94 ± 1.18 ^α^	3.72 ± 0.46 ^α^
PCL (wet)	9.44 ± 0.31	11.46 ± 1.86	1.96 ± 0.33
PCL/gelatin (wet)	10.07 ± 0.39	5.31 ± 0.89 ^β^	3.10 ± 0.32 ^β^

^α^ *p* < 0.05 compared with PCL (dry), ^β^ *p* < 0.05 compared with PCL (wet).

**Table 2:** Semi-quantitative analysis of green fluorescence area percentages in immunofluorescence (IF) staining image of calretinin and E-cadherin.

Culture Time (Days)	Calretinin (%)		E-Cadherin (%)	
	PCL	PCL/Gelatin	PCL	PCL/Gelatin
1	10.68 ± 0.57	23.13 ± 1.00 *	10.26 ± 0.35	28.10 ± 0.16 *
3	20.10 ± 0.30	49.88 ± 0.43 *	21.20 ± 0.36	36.82 ± 0.35 *
5	38.82 ± 0.33	80.20 ± 0.54 *	38.10 ± 0.20	66.20 ± 1.84 *
7	41.33 ± 1.00	88.10 ± 0.20 *	48.30 ± 1.45	84.70 ± 0.21 *

* *p* < 0.05 compared with PCL.

## Data Availability

The data presented in this study are available on request from the corresponding author.
